# Games and Creativity: A Theoretical Framework

**DOI:** 10.3390/jintelligence14020021

**Published:** 2026-02-02

**Authors:** Maxence Mercier, Samira Bourgeois-Bougrine, Todd Lubart

**Affiliations:** 1 KEDGE Arts School, KEDGE Business School, 75012 Paris, France; 2 LaPEA, Université Paris Cité and Université Gustave Eiffel, 92100 Boulogne-Billancourt, France; samira.bourgeois-bougrine@u-paris.fr (S.B.-B.); todd.lubart@u-paris.fr (T.L.)

**Keywords:** creativity, games, theoretical framework, game-based learning, experiential learning

## Abstract

This article introduces a theoretical framework centered on enhancing creativity through gaming, termed the Game-based Creativity Enhancement Framework (G-CEF). Rooted in experiential learning and game-based learning theories, the framework adopts an input–process–output paradigm: two inputs (personal attributes and game attributes), one process stage (learning situation), and outputs (learning improvements and acquisitions). Personal attributes take the form of conative dispositions and variables common to both creativity and games, which help explain why gaming habits and creativity are linked, particularly outside the laboratory. Six variables are identified and presented: playfulness, imagination, mind-wandering, mindfulness, psychological capital and motives. The second input corresponds to game attributes, which help explain why and how games can help improve creativity. Two forms of game attributes are presented: affordances and game mechanics. Eight types of affordances were identified: degree of flexibility, narrative, tools, environment, content creation, avatar, progression and replayability. Five types of game mechanics were also identified: originality, divergent thinking, convergent thinking, mental flexibility and creative dispositions. The learning situation within games represents a four-step cyclical experiential learning process: concrete experience, reflective observation, abstract conceptualization, and active experimentation. Lastly, the framework details enhancements in creativity due to gaming, supported by a literature review examining the impact of different game types on creativity.

## 1. Introduction

Creativity is arguably one of the most critical skills of our contemporary era (Kaufman et al., 2017). Throughout history, the capacity to generate novel and contextually relevant ideas has served as the driving force behind artistic masterpieces and societal progress (Archibugi et al., 2013). From Leonardo da Vinci’s visionary concepts to Marie Curie’s scientific breakthroughs, creative individuals have shaped the course of human civilization (Kaufman & Beghetto, 2009).

The significance of creativity extends far beyond its association with renowned figures. It permeates our daily lives, influencing both personal and professional spheres (Conner et al., 2018). In the professional sphere, creativity is associated with enhanced performance, innovation, and job satisfaction (Gong et al., 2019; Yee et al., 2014). Forward-thinking organizations recognize the value of fostering a creative work environment that encourages employees to think outside the box, explore new possibilities, and generate innovative solutions (Amabile, 1988, 2018). Moreover, creativity holds implications for personal well-being and fulfillment. Engaging in creative pursuits at home cultivates a sense of pleasure, self-expression, and fulfillment (Benedek et al., 2020). Whether it involves painting, writing, or cooking, creative endeavors provide an avenue for individuals to express their imagination, channel their emotions, and engage in meaningful self-expression (Kaufman & Beghetto, 2009; Silvia et al., 2014). The impact of creativity also extends to resilience and adaptability during challenging times. In the face of adversity and uncertainty, creative individuals possess the ability to think differently, find unconventional solutions, and adapt to changing circumstances (Orkibi, 2021).

Recognizing the potential for enhancing creativity, researchers and practitioners have developed various methods and techniques to nurture this skill (Sternberg, 2019). The common thread among these methods is the acknowledgment that creativity is not an innate talent but a trainable skill that can be honed through practice and experience. This paper adopts the view that effective creativity training and enhancement aligns with experiential learning.

In other words, creativity gains stem from active engagement and hands-on, concrete experience with creative activities. Furthermore, this paper argues that game-based learning constitutes an effective and cost-efficient operationalization of experiential learning. This is because games provide an engaging and stimulating environment, where learners can explore, practice and experiment with target skills. The main proposition of the paper is as follows: games constitute an effective and cost-efficient way to improve and learn creativity, through optimal experiential learning. As such, a comprehensive framework is introduced: the Game-based Creativity Enhancement Framework (G-CEF).

### 1.1. Creativity

Creativity is a complex and multifaceted construct that has captured the interest of researchers, educators, and practitioners across various disciplines. At its core, creativity involves the generation of novel and useful ideas, products, or solutions to problems (Sternberg & Lubart, 1999). Given its importance in driving innovation, problem-solving, and adaptability, understanding the nature of creativity and the factors that promote its development has become an essential area of inquiry in psychology, education, and management.

One influential model that offers a comprehensive understanding of creativity is the Four C Model, proposed by Kaufman and Beghetto (2009). This model categorizes creativity into four distinct but interrelated levels: mini-c, little-c, Pro-c, and Big-C creativity. Mini-c creativity refers to the personal insights and discoveries that individuals make as they engage in creative problem-solving or self-expression. Little-c creativity involves everyday problem-solving and the generation of novel ideas or solutions within familiar contexts. Pro-c creativity refers to the professional expertise and creative accomplishments demonstrated by individuals who have mastered a specific domain. Finally, Big-C creativity represents the groundbreaking, transformative contributions made by eminent creators, whose work has a lasting impact on their respective fields. The Four C Model underscores the importance of recognizing and fostering creativity at different levels and in various contexts, highlighting the idea that creativity is not limited to exceptional individuals but is a capacity inherent in everyone (Kaufman & Beghetto, 2009).

This perspective further aligns with the multivariate approach of creativity (Lubart et al., 2015). A multivariate approach to understanding creativity encompasses cognitive, conative/dispositional, affective/volition-related, and contextual factors. This approach emphasizes the interplay of these factors in shaping creative thinking and behavior.

Cognitive factors involved in creativity include the ability to engage in divergent thinking, which involves generating multiple, varied solutions to open-ended problems (Runco & Acar, 2019), as well as convergent thinking, which entails synthesizing diverse ideas, evaluating and selecting the most effective solution from a range of alternatives (Cropley, 2006). Creative thinking also requires the ability to make remote associations, draw on analogical reasoning, and shift between various perspectives or mental states (Ward & Kolomyts, 2010). Research on cognitive factors has also expanded to include the role of cognitive flexibility and executive functions in facilitating creative thinking (Benedek et al., 2014).

Conative or dispositional factors, such as personality traits have also been linked to creativity. For instance, factors such as openness to experience, tolerance of ambiguity, and self-efficacy have been associated with higher levels of creative thinking and behavior (Feist, 2010; Karwowski & Lebuda, 2016). Additionally, research has highlighted the role of cognitive style, or the preferred way individuals process information and approach problem-solving, in shaping creativity (Kozbelt et al., 2010).

Affective and volition-related factors, including motivation and emotion, play a significant role in creativity as well. Intrinsic motivation, or the inherent drive to engage in an activity for its own sake, has been consistently linked to enhanced creativity (Amabile, 1996; Hennessey & Amabile, 2010). Furthermore, specific emotional states, such as positive affect, can promote cognitive flexibility and the generation of novel ideas (De Dreu et al., 2008).

Contextual factors encompass social, cultural, and environmental influences that can shape creativity by either facilitating or constraining the expression of creative ideas and behavior (Glăveanu, 2010). For example, supportive and diverse environments that encourage risk-taking, collaboration, and the exchange of ideas can foster creativity (Amabile, 1996; Hunter et al., 2007). Moreover, cultural values, norms, and expectations can influence the perception, evaluation, and development of creative ideas and products (Glăveanu, 2010).

### 1.2. Creativity Enhancement

Creativity is increasingly important, valuable, and valued (Archibugi et al., 2013). It is equally important to examine if and how creativity can be enhanced. Creativity enhancement methods are designed to help people develop more of their potential. Different approaches exist and target various aspects of creativity (Haase et al., 2023): cognitive, social, attitudinal, motivational, environmental. Training creativity entails an understanding of the creative process and the acquisition of creativity-related skills and techniques enabling creative effort.

A recent meta-analysis (Haase et al., 2023), found that, across 332 effect sizes from 84 studies, creativity could indeed be improved and trained, furthering Scott et al. (2004) previous review. The effect size was medium (*g* = 0.53) and demonstrated that 70.09% of participants in experimental conditions were more creative than participants in control groups. Furthermore, 32% of studies found an effect larger than the average effect size found by Scott et al., of 0.68, establishing even more the malleability of creativity.

### 1.3. Experiential Learning

This paper proposes that most effective creativity enhancement methods are aligned with experiential learning theory. Experiential learning (EL) can be described as “the process whereby knowledge is created through the transformation of experience. Knowledge results from the combination of grasping and transforming experience” (Kolb, 1984, p. 41). Experiential Learning Theory (ELT) provides a holistic model of the learning process, notably in adult development. It emphasizes the central role of experience in the learning process. As such, ELT is distinct from cognitive learning theories that emphasize cognition over affect, and from behaviorist learning theories which deny any role for subjective experience (Kolb, 1984). ELT argues learning cannot occur without “hands-on” experience (Morris, 2020).

Learning occurs in a four-part cyclical process. First is concrete experience (CE) which asks learners to participate and engage in a new experience. Second is reflective observation (RO), which asks learners to gain a self-awareness of their cognitive and affective response to the new experience. Third is abstract conceptualization (AC), which asks learners to connect their old knowledge and experience to new thoughts, feelings, and experiences, in order to conceptualize situations from a fresh perspective. Finally, there is active experimentation (AE), when learners apply their new ideas to other experiences.

These four stages repeat themselves throughout the learning process, in both bottom-up and top-down dynamics. Specific experience is transferred to an abstract conceptualization upon reflection, which is then actively tested through new experiences. Immediate or concrete experiences serve as the basis for observation and reflection. These reflections are then assimilated and distilled into abstract concepts from which new implications for action can be drawn. Afterwards, these implications can then be actively tested and serve as guides for new experiences to come.

### 1.4. Game-Based Learning

Game-Based Learning (GBL) is an instructional approach that integrates digital or non-digital games into the learning process to engage learners and enhance their knowledge, skills, and motivation (Prensky, 2001). GBL leverages the intrinsic and extrinsic motivational factors of games, such as challenge, competition, and rewards, to foster a learning environment that is interactive, immersive, and adaptive (Plass et al., 2015).

The potential of game-based learning lies in its ability to motivate and engage learners. Games often include elements such as points, badges, leaderboards, and levels that create a sense of competition and achievement, thereby enhancing learner motivation (Kapp, 2012). They also provide immersive narratives and interactive environments that captivate learners’ attention and stimulate their imagination.

Game-based learning can facilitate both individual learning and collaborative learning. Single-player games can offer personalized learning experiences that adapt to individual learners’ needs and progress. On the other hand, multiplayer games can promote teamwork, communication, and negotiation skills as learners work together to achieve common goals (Plass et al., 2015).

Importantly, game-based learning offers learners opportunities for practice and repetition, which are essential for mastering new skills and knowledge. Well-designed games provide immediate feedback, allowing learners to recognize their mistakes, understand the correct responses, and adjust their strategies accordingly (Shute et al., 2009).

### 1.5. Integrating Experiential Learning and Game-Based Learning

Both approaches view learning as a dynamic process that requires active engagement and application. What is more, game-based learning provides a fertile ground for experiential learning. Learners/players can go through and experience the different stages of the experiential learning process and repeat this process thanks to the iterative nature of games.

#### 1.5.1. Concrete Experience

Concrete experience is the cornerstone of experiential learning theory. In EL, learners are involved, active and engaged participants in the learning process. This participation is central, as learning by doing cannot occur without it (Munge et al., 2018). It is a hands-on task-oriented process (Blair, 2016), based on direct experience, and thus requires active participation from learners. In the context of game-based learning, this stage involves learners physically and intellectually engaging with the game mechanics and dynamics. They interact with the game environment and also with other players, thus enabling a multifaceted experience (Blair, 2016; Jordan et al., 2018). Knowledge is situated in contexts, and interactions with the material and with other participants are key (Harper, 2018).

Learning involves risk, as EL incorporates novel and challenging experiences. The uncertainty and unpredictability inherent in games resonate with the risk-taking element of experiential learning (Davidson et al., 2016). The challenges posed by games require learners to step outside their comfort zones and confront novel experiences, which can already stimulate creativity (Cropley, 2006).

#### 1.5.2. Reflective Observation

Reflective Observation, the second phase of Kolb’s experiential learning cycle, involves introspective analysis and critical reflection on the experiences from the first stage (Kolb, 1984). This involves examining one’s own reactions and the situation’s consequences, thereby extracting insights from the concrete experience. This process of reflection allows learners to view the experience from different perspectives and gain a holistic understanding of the event (Kolb et al., 2001). In the context of game-based learning, this stage requires learners to reflect upon their actions, decisions, and outcomes within the game (Whitton, 2012). They scrutinize their strategies, interpret feedback, analyze their successes and failures.

Games tend to involve complex scenarios that require players to adopt different perspectives and approaches. This process allows them to identify patterns, question assumptions, and develop new insights (Moon, 2004). Furthermore, the social aspect of many games encourages players to reflect not only on their own actions but also on their interactions with other players (Steinkuehler & Duncan, 2008). Reflective observation serves as a bridge between practical experiences and the abstract cognitive processes of learning, allowing learners to transform their experiences into valuable insights that inform the next stages of learning.

#### 1.5.3. Abstract Conceptualization

The third phase in Kolb’s experiential learning cycle, Abstract Conceptualization, involves cognitive structuring of reflections into coherent, abstract concepts (Kolb, 1984). In this phase, the learner synthesizes reflections from the RO stage into structured theories, creating or modifying existing conceptual frameworks to make sense of their experience. This cognitive transformation of experiential insights into intellectual constructs allows learners to understand the underlying principles and relationships that govern the experienced event (Kolb et al., 2001). In the context of game-based learning, players draw upon their reflective observations and formulate theories or strategies that they can employ in the game. These theories involve novel approaches to overcoming game obstacles or achieving objectives. Games provide learners opportunities to and require them to understand complex systems and rules, pushing them to develop and refine their thinking skills (Squire, 2006).

This stage enables learners to generalize and apply their learning across different contexts. In games, learners also generalize their experiences and abstract theories, finding connections between game scenarios and real-world contexts (Garris et al., 2002). This process of abstraction promotes transferable skills that can be applied beyond the game environment.

#### 1.5.4. Active Experimentation

Active Experimentation, the final stage of Kolb’s experiential learning cycle, entails applying the abstract concepts formulated in the previous stage to new situations (Kolb, 1984). In this stage, learners test the validity and applicability of their abstract theories, refining further their understanding through the outcomes of these applications (Kolb et al., 2001). Within game-based learning, this phase involves the application of conceptualized strategies and solutions to new game scenarios or challenges (Whitton, 2012).

The iterative nature of games, with multiple levels or scenarios, allows learners to experiment with their strategies, adapt to new situations, and refine their understanding based on the outcomes. The dynamic and often unpredictable game environment fosters a culture of trial-and-error, which can encourage learners to take risks (Henriksen et al., 2021). Moreover, the collaborative nature of many games facilitates collective experimentation, where learners can test their solutions in a social context and learn from their peers’ strategies (Steinkuehler & Duncan, 2008). This stage enables a pragmatic approach to learning, encouraging learners to actively engage with their environment and learn from their interactions (McCarthy, 2010). Active experimentation concludes one cycle of learning, paving the way for the next cycle to begin with a new concrete experience.

#### 1.5.5. The Game-Based Creativity Enhancement Framework (G-CEF)

**Figure 1:**
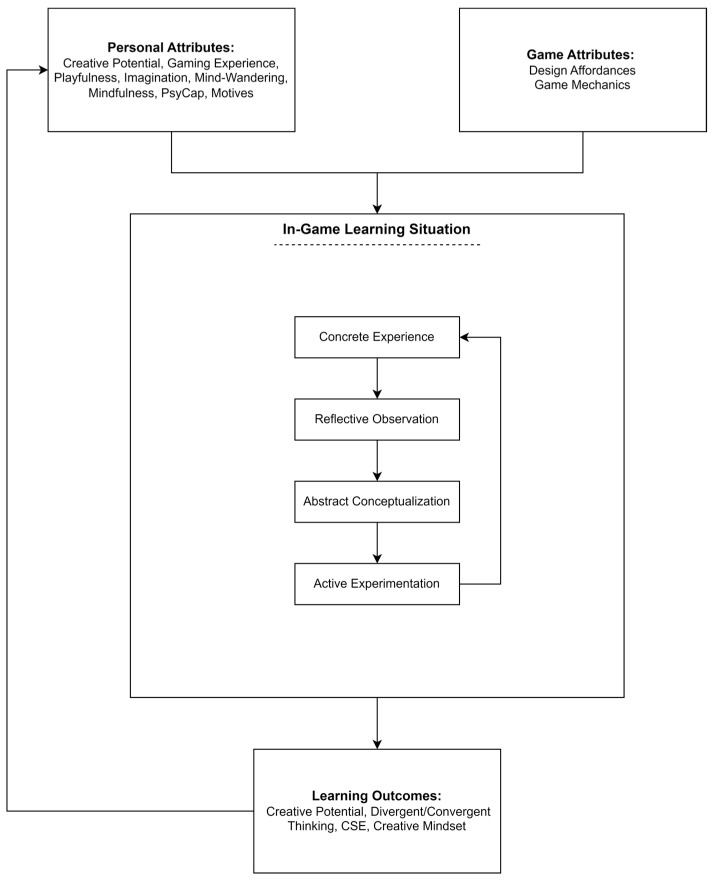
Game-based Creativity Enhancement Framework.

This paper aims to propose a comprehensive framework, based on experiential learning theory and game based-learning, while focusing specifically on creativity enhancement: the Game-based Creativity Enhancement Framework (G-CEF). In line with this objective, the framework adopts an input–process–output paradigm (Figure 1): two inputs (personal and game attributes), a process stage (the learning situation) and outputs (i.e., learning outcomes). The first input corresponds to the person attributes. Creativity is influenced by numerous factors: openness to experience, tolerance of ambiguity, imagination, etc. Personal attributes refer thus to dispositional constructs common to both gaming habits and creativity: playfulness, imagination, mind-wandering, mindfulness, psychological capital, and motives. Second are situational factors/game attributes. This second input refers to elements in games that are conducive to creativity and participate to improve or facilitate it. This includes game design affordances, and game mechanics. Three forms of games are included in the present framework: video games, board games, and role-playing games.

The learning situation integrates experiential learning and its four-stage process: concrete experience, reflective observation, abstract conceptualization, and active experimentation. This four-stage process corresponds to the actual game experience, and the learning processes which occur within. Finally, learning outcomes refers to different aspects and manifestations of creativity that are or could be improved upon through game play. This includes both short-term and immediate effects of game play, as well as potential long-term and transfer of learning.

## 2. Personal Factors

Several cross-sectional studies have linked creativity and gaming habits, examining various game types. Cross-sectional studies examining the relationship between video game play and creativity have produced mixed findings. On one hand, Jackson et al. (2012) and Blanco-Herrera et al. (2019) found significant positive correlations between video game play and indicators of divergent thinking and graphic creativity, respectively, suggesting that habitual engagement in video games may contribute to the development of specific dimensions of creativity. Similarly, Mercier and Lubart (2023b) conducted a questionnaire-based survey of 370 workers and found that playing video games was associated with higher creativity in the workplace. This association was fully mediated by optimism. On the other hand, Čábelková et al. (2020) reported a negative correlation between video game play time and emotional creativity, indicating that excessive video game play may be associated with decreased emotional creativity.

Cross-sectional research on role-playing games has also shown associations with creative outcomes. Chung (2013) compared the creativity of tabletop role-playing game players, online role-playing game players, and nonplayers. Results showed that tabletop gamers were more creative than non-gamers, with online gamers falling between the two groups. This finding implies that engagement in role-playing games, particularly in their tabletop form, may be associated with enhanced creativity.

Lastly, preliminary evidence has appeared regarding board games. Mercier and Lubart (2023a) found significant positive correlations between the frequency of playing board games and various indicators of creative potential, such as openness, originality in divergent thinking, creative self-efficacy, and creative personal identity. A subsequent study (Mercier et al., 2025) showed further evidence of this link, with significant correlations between board game play habits and two aspects of creative potential: creative self-concept and creativity in the workplace. These findings imply a potential link between habitual board game play and the enhancement of creative potential.

Here we propose several common denominators that could explain the links between gaming habits and creativity: playfulness, imagination, mind-wandering, mindfulness, psychological capital, and motives.

### 2.1. Playfulness

Playfulness can be understood as an individual difference variable that allows people to frame or reframe everyday situations in a way to experience them as entertaining, intellectually stimulating and personally interesting (Proyer, 2017). They have a preference for complexity over simplicity, and for unusual activities, topics and persons (Proyer et al., 2019). Furthermore, they are capable of using their playfulness under difficult conditions to resolve tension. Early playfulness research focused predominantly on children (Guitard et al., 2005), leading to sporadic research for adults. This could be explained because of two perceptions. First, social manifestation of playfulness can be seen as less acceptable among adults (Lieberman, 1977). Second, playfulness seems to lack apparent practical usefulness (Olsen, 1981). However, playfulness is ubiquitous among adults as much as it is in children (Sutton-Smith, 1966), and seems to be even more permeating as we grow older. The forms of playfulness behaviors expand from sensorimotor play to more frequent social, imaginative and cognitive play (Lieberman, 1977). The expression of playfulness increasingly crosses the boundaries of work and leisure, and “extends to all life situations” (Guitard et al., 2005, p. 19).

Playfulness is explicitly linked to games, as playful individuals seek the kind of activities that games provide (Proyer et al., 2019), however no empirical results yet support this theoretical assertion. Additionally, playfulness is also linked to creativity. Proyer (2012), in a study of students (*N* = 212), observed that playfulness was correlated to self-rated creativity (*r* = 0.26). However, it was not correlated with either fluency or originality. The links between playfulness and creative activities were also examined (Proyer et al., 2019) using the K-DOCS (Kaufman, 2012), a measure of creative behaviors in five domains. Focusing on adult employees, playfulness was associated with everyday creativity (*R*^2^ = 0.18), performance creativity (*R*^2^ = 0.24) and artistic creativity (*R*^2^ = 0.26). A recent cross-sectional study (Mercier et al., 2025) investigated the role of adult playfulness as a mediator between board game play habits and two aspects of creativity: creativity in the workplace and creative self-concept (i.e., the confidence in one’s ability to be creative and the importance one attaches to creativity in their life; Zandi et al., 2022). Results showed that adult playfulness fully mediated the relationship between board game play habits and creativity in the workplace and partially mediated the relationship board game play habits and creative self-concept.

### 2.2. Imagination

Imagination is another construct that can be associated with both games and creativity. Imagination refers to the “formation of ideas, images and concepts in one’s mind’s eye” (Zabelina & Condon, 2020). It is conceptually close to mental imagery, which refers to the extent to which one is able to generate a sensory experience from information stored in memory (Kosslyn et al., 2001). Imagination has long been construed as the ability to conjure such mental imagery, that is, visualization in the absence of stimuli (Pearson et al., 2015). However, Zabelina and Condon (2020) argue that imagination encompasses a wide range of sensory modalities and affects an equally wide range of outcomes. Imagination thus refers broadly to the capacity to construct that which is not currently present to the senses (Gotlieb et al., 2019). We engage in it intentionally and unintentionally, solitarily, and collectively.

Imagination can be seen as the source of creativity (Gotlieb et al., 2019). Imagination lays the foundations for and is important to creativity. One notable form of imagination is social-emotional imagination, broadly defined as the ability to conceive and reflect on various social perspectives and scenarios, and their implications (Gotlieb et al., 2016). The manifestations of this form of imagination, such as perspective-taking, support individuals’ and groups’ creativity by helping them understand the variety of experiences and situations, inside and outside themselves (Sawyer, 2017; Taylor et al., 2003), and appreciate the diversity of views and approaches to problems (Hoever et al., 2012).

Individuals need enriching environments to provide fodder for their imagination, to produce creativity. We argue that games can help nurture and nourish imagination, notably through exposition to worlds and stories. Hayes et al. (2015) argued imagination was the catalyst that links abstract consciousness to the generation of material experience. Through imagination, individuals become immersed in a space of play in which possible worlds and selves can be imagined. Video games offer a great opportunity to enable the presentation of imaginary scenes and help transport players to different worlds and spaces, thus enriching imagination (Cheng, 2021). Role-playing games are particularly apt in this regard. Role-playing games are games in which players create and embody an imaginary character, who then acts and moves in a defined world and in situations created and narrated by a game master (Karwowski & Soszynski, 2008). By their very nature, role-playing games involve a notion of shared fantasy, engaging individual imagination, through the guidance of the game master (Hughes, 1988).

One particularly relevant area of research on imagination and creativity concerns the study of the links between pretend play and creativity (Russ & Doernberg, 2019). Pretend play is a symbolic behavior in which “one thing is playfully treated as if it were something else” (Fein, 1987, p. 25). For example, a piece of wood becomes a sword, or a shoelace becomes a snake. At its core, pretend play represents a natural form of creativity in childhood (Fein, 1987). Pretend play represents a vehicle for the expression of many processes important in creative production (Russ, 2016), as well as a vehicle for practicing with ideas and images (D. G. Singer & Singer, 2009). In pretend play, children use objects to represent other objects, make up stories, use fantasy and role-play, feel and express affects. Play introduces the child to the feeling that accompanies creative expression.

There has been notable focus on the relation between play and divergent thinking, the ability to generate multiple ideas or solutions (Guilford, 1967). Play is practice with divergent thinking (J. L. Singer & Singer, 2005). D. L. Singer and Rummo (1973) found a relation between play and divergent thinking in kindergarten boys; Wyver and Spence (1999) found the same results in preschoolers. There have been longitudinal studies, showing that pretend play predicts divergent thinking four years later (Russ et al., 1999; Wallace & Russ, 2015). Notably, Wallace showed components such as affect expression and narrative organization were associated with creativity over time. Fehr and Russ (2016) found correlations between pretend play and creative storytelling in preschoolers, and Hoffmann and Russ (2012) found similar results in primary school students. Therefore, there seems to be good evidence for the link and its stability over time (McVay et al., 2025; Russ & Doernberg, 2019).

### 2.3. Mind-Wandering

Creativity can stem from seemingly contradictory processes (Agnoli et al., 2018). It can occur from lack of executive control over one’s mental activity, which could be described as getting lost in one’s own thoughts; in other words, mind-wandering. On the other hand, it can be the awareness of one’s own thoughts, affects and sensory experiences, as well as the exclusion of distractions that can lead to finding creative solutions and ideas; this is mindfulness. This part will focus on the first phenomenon, mind-wandering. Mind-wandering occurs when attention drifts away from an on-going task and external environment towards internal thoughts unrelated to the task, such as memories and prospective thoughts (Smallwood, 2013). This experience can also be described as “having one’s attention shifting away from the objective world and its related perceptual input, and towards internal reflection” (McMillan et al., 2013).

Mind-wandering has been associated with maladaptive outcomes, such as reduced executive and attentional controls, but it can also be seen as a valuable cognitive capacity. Notably, mind-wandering might help better one’s performance when occurring during an incubation period (Baird et al., 2011). Links between mind-wandering and creativity can be explained by an increase in unconscious associated processing, which produces a spreading activation conducive to higher creativity (Baird et al., 2012). However, results have sometimes been contradictory (Hao et al., 2015), which can be attributed to the unidimensional view of mind-wandering as involuntary. Mind-wandering should instead be decomposed in deliberate or spontaneous forms. That is, whether it emerges spontaneously or, somehow, under the individual’s mental control. In deliberate mind-wandering, attention is intentionally shifted from the focal task towards internal thoughts (Chiorri & Vannucci, 2017), as opposed to the uncontrolled shift observed in spontaneous mind-wandering. Agnoli et al. (2018) showed that deliberate mind-wandering was a positive predictor of creativity (notably originality), whereas spontaneous mind-wandering was a negative predictor instead. The control over mind-wandering can be considered a central element in the creative production, as it may be increasing originality by increasing new and unrelated thoughts into the thinking process (Preiss et al., 2020).

Mind-wandering intersects with the realm of games through the concept of Game Transfer Phenomena (GTP). GTP refers to the involuntary carryover of game elements, images, and experiences from virtual environments to the real world, usually taking the form of sensory or cognitive intrusions, blurring the line between game-related mental content and daily life (Ortiz de Gortari & Diseth, 2022; Ortiz de Gortari & Griffiths, 2016). This phenomenon can be seen as a manifestation of mind-wandering, where players’ attention shifts from the external environment to their internal thoughts, often containing elements of gameplay, even when not actively engaged in gaming. This phenomenon occurs in virtually all players. As shown in Ortiz de Gortari & Griffiths’ descriptive analysis (Ortiz de Gortari & Griffiths, 2015), 96.6% of video game players reported having experienced GTP, and the majority reported having experienced it more than once (95.3%).

### 2.4. Mindfulness

In contrast to mind-wandering, there is mindfulness. Mindfulness refers to a state of conscious, sustained, and focused awareness resulting in a nonjudgmental attention to the present moment (Tang et al., 2015). This concept stems from the Buddhist meditation tradition, which suggests this state can be reached and improved upon through meditation. Mindfulness is a non-elaborative, present-centered awareness where all thoughts and feelings are acknowledged and accepted (Bishop et al., 2004). In mindfulness, attention is regulated such that increased awareness is brought to the current field of thoughts, feelings, and sensations, while additionally being immersed non-judgmentally in the present moment. This specific state of mind can be very useful in disengaging individuals from automatic thoughts and habits (Baer et al., 2004).

Early studies on the links between mindfulness and creativity have produced mixed results. Ostafin and Kassman (2012) observed that mindfulness benefitted the ability to solve insight problems. This might be due to the reduced tendency to rely on habitual responses when searching for a solution. In contrast, Zedelius and Schooler (2015) found opposite results, i.e., a decrease in performance on insight problems. These contradictions are probably due to the multidimensional nature of mindfulness. It is composed of five dimensions: observing, acting with awareness, describing, being non-reactive, and not judging. Drawing on this multidimensional approach, Baas et al. (2014) found very consistent findings, as only the ability to observe and attend to the stimuli (observing) predicted creativity, across a series of four studies. In a study by Agnoli et al. (2018), results indicated that the ability to describe one’s feelings and beliefs with words (describing) was the main predictor of creative achievements, as measured by the CAQ (Carson et al., 2005). On the other hand, the ability to not react to inner experiences (being non-reactive) was negatively associated with originality. Originality is associated with the ability to feel and recognize intense new feelings that might be externalized, especially so in the arts (Silvia, 2005). Mood states evidently affect creativity; being non-reactive to such states blocks their beneficial effects on originality. In the context of workplace creativity, overall mindfulness has been shown to be beneficial to employee creativity (Khan & Abbas, 2022), yet the relationship seem to be culture-dependent: whereas positive emotions mediate this relationship in the United States, negative emotions appear to be the predominant mediator in the case of the Philippines or Turkey (Gip et al., 2022).

Mindfulness can be linked conceptually to flow, a mental state occurring when there exists a balance between challenge and skills, which includes awareness, a sense of calm control and absorption in the present activity (Engeser et al., 2021). Gackenbach (2008a) found frequent players reported more flow experiences than infrequent or non-players. Sherry (2004) argued that games, particularly video games, possess ideal characteristics to create and maintain flow experiences, which can be extrapolated to mindful states as well. Gackenbach (2008b) likened games to a form of meditation practice. Due to the absorption in meditation, mindfulness should be associated with playing games, through immersion and/or absorption.

### 2.5. Psychological Capital

Psychological Capital (PsyCap) has long been associated with creativity, notably in the workplace. PsyCap is a higher-order construct that can be referred to as a person’s positive psychological state (Luthans et al., 2007). PsyCap is characterized by four components: “(1) having confidence (efficacy) to take on and put in the necessary effort to succeed at challenging tasks; (2) making a positive attribution (optimism) about succeeding now and in the future; (3) persevering toward goals and, when necessary, redirecting paths to goals (hope) in order to succeed; and (4) when beset by problems and adversity, sustaining and bouncing back and even beyond (resiliency) to attain success” (Luthans et al., 2015, p. 4). Several studies found links between creativity and overall PsyCap. For instance, Sweetman et al. (2011) found that overall PsyCap predicted divergent thinking over and above the four individual components. This finding was observed as well for self-reported creativity (Cai et al., 2019). Using a component-focused approach, Yu et al. (2019) found that components contribute in different ways to creativity, with hope and optimism as the strongest predictors, and resilience acting as a mediator between these two components (hope and optimism) and creativity.

Similarly, PsyCap and its components can be linked to games. Adversity is a core aspect of games, and notably video games. To face setbacks and difficulty, a specific mindset is necessary. Failure or loss is commonplace and often encountered while playing most games, and thus hope and resilience are often required, else one would usually quit easily. The importance of defeat can also be reflected in another component of PsyCap, optimism. To persevere through adversity, an optimistic style is essential. There may be a temptation to give up when playing games and experiencing failure. In such situations, being optimistic can prove helpful, as an optimistic style enables one to see negative events as temporary and thus surmountable, even more so when such efforts usually end in rewarding outcomes.

Mercier and Lubart (2023b) conducted a questionnaire-based study, examining the links between video games and creativity, with a potential mediation via PsyCap explaining this relationship. Their findings show a full mediation of the link between playing video games and workplace creativity, through optimism: playing video games was positively associated with higher optimism, which in turn was associated with more creativity in the workplace.

### 2.6. Motives

What motivates people to engage in creativity (and in games)? Creativity has been consistently linked to intrinsic motivation, suggesting creative work is often interesting and satisfying in itself. This does not imply the contrary with extrinsic motivation, as they can act independently (Amabile et al., 1994). However, overall mixed results have been found for extrinsic motivation (Hegarty & Plucker, 2012). Beyond these two types of motivation, one can wonder as to what exactly motivates people to pursue creative endeavors. Benedek et al. (2020) conducted a comprehensive review of the literature, in which they identified nine specific motives for creativity: enjoyment, expression, challenge, coping, social, prosocial, recognition, material, and duty. First is enjoyment, that is, doing something because it is enjoyable, fun, pleasurable (Amabile et al., 1994). Expression refers to the motive to express one’s feelings, emotions or thoughts towards others or oneself (Gould et al., 2008; Hegarty, 2009). Challenge corresponds to the desire to achieve something, to expand one’s skills or knowledge, and to prove one’s competence (Beard & Ragheb, 1983; Manfredo et al., 1996). It is akin to the need for competence (Ryan & Deci, 2000). Coping corresponds to the need for tension reduction and the desire for emotional calm (McGuire, 1974; Reiss, 2000). The social motive refers to the wish to spend time with others and be close to them. This is one of the most consistent motives (Tay & Diener, 2011) and one that is akin to the need for relatedness (Reiss, 2000; Sheldon et al., 2001). The prosocial motive is an altruistic aim to help and/or bring pleasure to others (Auger & Woodman, 2016; Beard & Ragheb, 1983). Recognition refers to the desire to gain acceptance, acknowledgement or even admiration from others (Reiss, 2000; Sheldon et al., 2001). The material motive means carrying out activities in pursuance of monetary rewards and/or material compensation (Gould et al., 2008). Finally, duty corresponds to the general feelings of responsibility or obligation, the feeling that “something has to be done” (Bernard et al., 2008; Ryan & Deci, 2000).

These nine motives for creativity could, for the most part, be equivalent to the motives that people possess for playing games. Early on, Beard and Ragheb (1983) identified common motives for leisure activities, such as games: intellectual challenge (challenge), social contact (social and prosocial), the experience of competency (challenge) and the wish to relieve stress and tension (coping). Enjoyment is also the evident and primary motivation behind playing games (Cheah et al., 2022; Sweetser & Wyeth, 2005). Expression can also be driving some individuals to play, notably in the case of sandbox games. Challenge is a core aspect of games, and thus a main motivation for people who like playing games of all sorts (Cheah et al., 2022). Coping is also present in many gaming habits, as playing soothing games can help reduce tensions and promote well-being (Barr & Copeland-Stewart, 2022; Pearce et al., 2022), and more violent games could be seen as cathartic as well (Lee et al., 2021). The social motive is one that is shared by many who play massively multiplayer online role-playing games (Cheah et al., 2022; Cole & Griffiths, 2007). Furthermore, games provide an opportunity to reunite friends around a board game or play with long-distance friends in online video games. Prosocial motives are best exemplified in people who opt to play support or healing roles in games, notably video games. Recognition can be seen in players who engage in highly competitive games. Material and duty motives, although rare, could also be construed as motives to play games. Esports (electronic sports) are one such example, referring to competitive video gaming where teams or individuals compete against each other in a video game (Bányai et al., 2020). This is highlighted by the colossal expansion of high school esports developmental programs (e.g., Hennick, 2019) and the high earning potential of esports players, in terms of salaries, cash prizes and sponsorships (Bányai et al., 2020; Newzoo, 2019).

## 3. Game Attributes

Three types of games are included in this framework: video games, board games and role-playing games. Video games refer to electronic games that involve human interaction with a user interface, generating visual feedback on a screen. They encompass a wide variety of genres, platforms, and interactivity levels. Video games are characterized by goals, challenges, rules, and user-generated inputs that affect on-screen outcomes (Li, 2008; Tavinor, 2008). A board game is a game with a certain number of rules that limit the possibilities, that requires a physical medium (e.g., a board, cards, dice) and that is played by two players or more (Gobet et al., 2004). Role-playing games are narrative-driven storytelling games where players assume the roles of characters in a fictional setting, directed and narrated by a game master (Hughes, 1988; Karwowski & Soszynski, 2008). Actions and outcomes are determined via the combination of character decisions, rules, and dice rolls, enabling players to co-create and navigate through narratives.

This section is divided into two parts. The first one focused on how games can provide opportunities to be creative, inside and outside of the game. These affordances can help explain the links between gaming frequency of play and creativity. As many games can provide such affordances, people who play more games encounter them more often, in turn potentially affecting their creativity. The second part focuses more specifically on games that are explicitly beneficial for creativity. That is, how particular games target, solicit and require some aspects of creativity within their mechanics, in turn improving creative potential outside of the game.

### 3.1. Design Affordances

Many definitions for affordances have been proposed over the years, from Gibson’s (1977) actionable properties of an environment, to Cardona-Rivera and Young (2013) simpler definition as opportunities for action, or Kim and Shute’s (2015) opportunities for a certain behavior. In many cases, affordances are contextually defined and domain specific. In this paper, we will use Hall et al. (2021) definition of affordances, which corresponds to the opportunities provided by a game for creative actions, behaviors, or thoughts.

Affordances play a key role in creative behavior, as interactions with the situation or environment can affect creativity (Amabile, 1996). Glăveanu (2013) argued that affordances can either allow or constrain creativity, as a creative individual can utilize available affordances in a novel manner. Cardona-Rivera and Young (2013) proposed a theory of affordances in games and identified three types: (1) real affordances, relating to actual game actions; (2) perceived affordances, relating to what a player perceives as possible in the game; (3) feedback affordances, relating to perceptual information targeted at promoting real affordances. The relation between perceived and real affordances is affected by wider factors, as a player’s experience, history and beliefs will affect how they perceive affordances in a specific game. For instance, having played a similar type of game previously might affect the extent to which they perceive affordances.

Multiple forms of design affordances for creativity in digital games have been identified by Hall et al. (2020, 2021). Namely, they identified eight specific affordances: degree of flexibility, narrative, tools, environment, content creation, avatar, progression, and replayability. In this paper, we argue that these affordances also apply to board games and role-playing games.

#### 3.1.1. Degree of Flexibility

This encompasses affordances in relation to autonomy over the play trajectory: having different routes, side quests, proposing different playstyles. This corresponds to what a game affords in terms of how the player can choose to play, and how accessible it is to test the boundaries imposed by the developers. Games with a degree of flexibility usually are those that propose task flexibility, in which you can complete challenges in multiple ways. Flexibility can be achieved through the alteration and/or modification of characteristics, abilities, to enable various valid play styles. This affordance ties into the possibility thinking aspect of creativity (Caillois, 2001), and involves experimenting through play, and asking “what if” (Craft et al., 1997).

Non-linear games are the main candidates, as they offer a variety of routes, and opportunities for diverging from the main narrative. Open-world video games allow players to create their own path through the game and define their routes. They often are the most engaging games, and those who grant the highest sense of achievement.

#### 3.1.2. Narrative

Narrative affordances relate to opportunities for creative engagement with the game’s narrative. Namely, how the story and characters prompt emotional engagement, reflection, and further exploration of challenging issues.

They characterize video games with strong narratives but can also occur in MMOs and non-narrated video games, in which players uncover the story and lore through gameplay, or even create their own personal narrative. Open-world games without a voiced protagonist can also provide more creative room and let the player engage and bring more to the story. For instance, choice-based dialogues give opportunities to make decisions that can affect the unfolding of events. This allows players to make meaningful choices and provide further opportunities for narrative exploration.

#### 3.1.3. Tools

This refers to affordances for the use of different types of tools. They relate to the variety of items, abilities, and range of movements. They add to the customization of the gaming experience and offer possibilities for problem-solving and potential creative expression within the game. They are often a precursor for task flexibility, as they allow challenges and missions to be solved and completed in different ways. Put simply, the more tools available, the more combinations and ways to solve ill-defined problems and challenges.

They can also take the form of alternative modes of transport in video games and an extended range of movements (e.g., swimming, climbing), which can encourage the player to explore its surroundings and the world at large. Tools should be useful: useless or weak tools might not help and thus not be used for progressing. However, they can potentially be used for personal challenges, for example winning the game with the weakest weapon available.

#### 3.1.4. Environment

This refers to opportunities to both explore and interact with the environment, such as utilizing terrain and environmental objects. Exploration is not sufficient, as interaction with the environment and objects is also important. Such affordances allow for an active experience and the possibility for discovering different game aspects. This is linked to the notion of ludic space (Aarseth, 2012). Games contain both a ludic space, referring to the playable area, and an extra-ludic space, the surrounding space the player cannot explore. In a linear game, most of the space is extra-ludic, whereas the opposite is true in open-world games. Extending this ludic space might provide greater opportunities for creative behaviors.

Open-world video games, in which the player is not restricted to a predetermined path, are particularly tailored to this form of affordances. The aesthetics of the game can also facilitate creative inspiration, inside and outside the game.

#### 3.1.5. Content Creation

This type of affordances refers to opportunities for creating in-game content, like building and crafting. Furthermore, they include possibilities for creating and integrating mods (modifications which add custom items, levels, characters, objects and interfaces) and add-ons. The possibility of creating allows for in-game occurrence and manifestation of little-c creativity. They provide opportunities to pursue one’s curiosity, through exploration and experimentation.

Sandbox and simulation video games are the ideal candidates, as they allow players to tailor the game experience to their own goals, creating “anything” they wish within the game. This can also take the form of creating your own maps and levels. Finally, games that are open to mods can add functionalities and customize further the experience. Having functional yet customizable items also participates in the engagement within the game: decoration is interesting, but interaction is preferable as it can help provide a more realistic experience.

#### 3.1.6. Avatar

This refers to possibilities for customization of the character appearance, personality, dialogue options and classes. This can become an additional goal within the game itself.

Different dialogue options can enable the exploration of different selves and personalities (Klimmt et al., 2009), sometimes clashing with one’s habitual behaviors and attitudes. This can take the form of appearance modifications: clothes, hairstyles, physique, voice. Such options and liberty can lead to portrayals that can either be faithful or weird, which can be an outlet for personal expression.

#### 3.1.7. Progression

This kind of affordance is indirectly linked to creativity, as it is related to aspects that help maintain player motivation. For example, progression provides a scaffolding mechanic that helps balance challenges, in line with the development of a player’s skills. The presence and achievements and their completion can also help in that sense, as skill learning becomes essential to complete successfully challenges, to bring a sense of self-efficacy and competence.

#### 3.1.8. Replayability

Replayability refers to what possibilities for replay there are: different endings, different playstyles, allowing for the experiences of different playthroughs. This refers also to the renewal of gaming experience: updates, patches, extensions. They provide opportunities for further engagement and help maintain motivation as well. Replayability is determined by the structure of the game (open vs. linear) and the structure of the narrative (divergent vs. linear). Games with different endings and divergent narratives enable players to explore the game differently. Such affordances allow the player to envisage and imagine possible outcomes to the story (Cardona-Rivera & Young, 2013), in turn encouraging creativity.

This can take the form of games that encourage players to replay them to discover the outcome of different actions, making each playthrough a unique experience. Linear games might provide less opportunities for renewal of gaming experience, but this can still happen in the case of different endings. This can also take the form of DLCs and add-ons that provide new storylines or maps, and thus further possibilities for exploration.

### 3.2. Game Mechanics

This section is based on the 4Cs Evaluation Framework for Games (Thornhill-Miller et al., 2023), which is itself based on the 4Cs framework, consisting of four 21st century skills: creativity, critical thinking, communication, and collaboration. The focus here will be on creativity specifically. The evaluation grid assesses a game’s effectiveness for training and improving creativity, based on the mechanics directed towards the practice and realization of this skill. If a game involves and requires creativity to a considerable degree to perform well, it will be considered as helpful and effective to improve creative potential.

The grid is based on the multivariate approach of creativity (Lubart et al., 2015), and is composed of five factors, among which four cognitive factors and one conative factor: *originality*, *divergent thinking, convergent thinking*, *mental flexibility*, and *creative dispositions.*

*Originality* involves generating ideas that are new, rare, or unexpected in relation to the ideas of other players, what one would naturally expect, and one’s own previous ideas. Video games like “Minecraft” and “Scribblenauts”, board games such as “Dixit” and “Codenames”, and role-playing games like “Dungeons & Dragons” all encourage players to think outside the box and come up with new and unique strategies or solutions. These gaming environments should foster creativity by continuously challenging players to develop novel ideas and expand their imaginative capabilities.

*Divergent Thinking* refers to the ability to generate many different ideas, explore multiple solutions to a problem, break free from conventional thinking, and explore various possibilities. Video games like “Besiege”, board games like “Imagine” and role-playing games such as “Fiasco” encourage divergent thinking by challenging players to think broadly and generate numerous ideas or approaches. These games often involve open-ended problems and require players to experiment with different strategies, leading to an expansion of divergent thinking.

*Mental Flexibility* encompasses changing perspectives on a problem, understanding it from different angles, detaching from initial ideas, generating ideas from diverse categories, and shifting between modes of thinking. Puzzle-based video games like “Portal” and “Braid”, as well as board games like “Hanabi” and “Pandemic”, require players to adapt their thinking and strategies based on the game’s evolving dynamics. These games often involve complex and shifting situations that demand continuous adaptation and the ability to think in multiple ways, promoting flexibility and adaptability.

*Convergent Thinking* involves observing similarities between different approaches to a problem, joining multiple elements to create a new idea, connecting seemingly unrelated elements, combining ideas with a common mediator, and evaluating ideas to select the best or most creative one. Board games like “The Big Idea” and video games such as “Kerbal Space Program” promote convergent thinking by helping players refine their ideas and solutions by synthesizing and integrating diverse perspectives or concepts. These games enhance players’ ability to find connections, make sense of complex situations, and ultimately develop more creative solutions.

*Creative Dispositions* relate to being open to new experiences and ideas, trying unknown things, taking calculated risks, and tolerating ambiguous situations, ill-defined problems, and contradictory information. Open-world video games such as “The Legend of Zelda: Breath of the Wild” and role-playing games like “Pathfinder” encourage creative dispositions by fostering a mindset that embraces experimentation, exploration, and learning, and by giving ill-defined problems and numerous possible trajectories. Players are thus more inclined to take risks and push their boundaries.

## 4. Learning Situations: Illustrations with Board Games

Learning cannot occur without experience. Learners, if they are to be effective, must be able to: (1) involve themselves fully and openly in new experiences, (2) reflect on and observe their experiences from many perspectives, (3) create concepts that integrate their observations into logical “theories”, and (4) use these theories to make decisions and solve further problems. Game-based learning provides players with an optimal and engaging environment for learning, allowing them to experience the experiential learning process and its stages, and repeat this process thanks to the iterative nature of games

To understand how the game-based experiential learning process takes place regarding creativity, let us take two examples derived from existing board games: Time’s Up, and The Big Idea.

### 4.1. Time’s Up

Time’s Up is a fast-paced, charade-based guessing game for a minimum of four players, divided into teams. The game proceeds in three rounds, each with a different method of hint-giving, using the same set of name cards: people, places, books, movies, etc. In the first round, players give unlimited verbal clues to their team, trying to get them to guess the name on the card, without saying any part of the name. Each team has 30 s to guess as many cards as they can, then plays moves to the next team. Once all cards are used, they are reshuffled for the next round. The second round involves players giving only one-word hints. If the team guesses correctly, they can guess again until their time runs out. The third and final round involves charades. Clue-givers must act out the name on their card without using any verbal clues. Teams guess within their 30 s interval.

“Time’s Up” involves several game mechanics conducive to creativity, for both the clue-giver and the guesser. The clue-giver must find ways to express the concept, in a non-explicit way. As such, they need to find novel ways to describe it. Furthermore, these clues must be relevant and adapted towards the guesser. If the idea is too remote from the guesser’s frame of reference, too random or not elaborated enough, the players will not advance or perform well. On the other hand, the guesser must make associations and generate ideas from the clues given to them. They need to be flexible and make associations and analogies, in a similar way to insight tasks. Finally, they need to converge into the correct answer, based on this process.

The concrete experience corresponds to the discovery of the game mechanics and the other players. The clue-giver receives the cards and tries to find ways to express indirectly the concepts that are. This first exercise can be arduous, even more so when the clue-giver and the guesser do not know each other. After the first 30 s, the second team gets to play. There, the clue-giver reflects on what worked and what failed and observes the other team as they play. For example, the clues given might be too “out there” for the guesser, or in contrast too straight forward and unoriginal, making it hard to make associations and find the concepts. Then, abstract conceptualization comes into play, as the clue-giver tries to adapt more to the guesser, finding strategies that better fit their duo. After the opposing round, the clue-giver gets to play once again, and apply the strategies that were generated in the meantime. If the clues were too straightforward, the player might try to be more original. Alternatively, they can adapt to the references and culture of the partner. This cycle goes on, up until the end of the game.

### 4.2. The Big Idea

The Big Idea is a party game designed for 3–6 players, in which they take the roles of inventors, creating new products to pitch to an audience. In it, players need to come up with products based on cards and then present and sell their inventions to the rest of the players. At the beginning of each round, players get six cards: three “Thing” cards and three “Qualifying” cards, that must be combined to create an invention. Each player is free to use as many cards as he or she wishes and in any order they see fit, if they can provide meaning for the invention. The inventors then give a persuasive pitch to the other players, describing what their product is and why it is great. Once every player has pitched their ideas, they vote for the best invention. A second round then starts, and so on. In The Big Idea, players need to find ways to combine seemingly unrelated cards and concepts, in a manner that is meaningful and compelling. The game is divided into two creation phases: the invention itself, then the pitch and its arguments.

The concrete experience again starts with the discovery of the game mechanics and materials. Here, this involves facing the six cards and their potential randomness and remoteness to each other. They then must find ways to connect them and select the best invention. Then, the player comes up with a pitch, to sell their product to the other players. After everybody is done working on their invention, the player begins to pitch their invention. Afterwards, the player listens to the other players’ pitches and inventions. During this part, the player engages in reflective observation, as they reflect on what they could have done better, compared to the ways the other players used their cards. They then enter abstract conceptualization, to come up with theories and strategies for what to do next turn. For example, if the player only used two cards out of the six available, they might want to try combining more cards. If their invention was interesting but the pitch did not sell well, they may be inspired by stories proposed by the better pitchers of the group. Once everybody has voted, players get new cards, with new possibilities of invention. At this point, active experimentation starts and players try to implement what they derived from the last step. This process repeats itself, as long as players are engaged in the board game.

## 5. Learning Outcomes

### 5.1. Board Games

Experimental and quasi-experimental studies have demonstrated the potential of board games for fostering short-term learning and improvement of creativity. Park and Lee (2017) found that incorporating board game play into the curriculum of two fifth-grade classes led to significant improvements in mathematical creativity, particularly when students could engage in free-play. Gonzalo-Iglesia et al. (2018) explored the impact of introducing commercial board games related to university course content on motivation and creativity. Their findings indicate that students who played these games reported higher motivation to learn and an enhancement in their creativity. Chen et al. (2021) employed a similar approach, by introducing a board game as teaching material in a high-school chemistry course. Results showed that such a method led to increased creative problem-solving skills from the students, notably in solution-finding. These findings highlight the potential of integrating board games into educational settings to support both motivational and creative outcomes.

In Mercier and Lubart’s (2023a) experimental study, they compared the effects of playing creative board games, non-creative board games, and a no-game control condition on participants’ originality. The results showed a significant, medium-sized improvement in originality after playing creative board games compared to the other two conditions. This finding suggests that board games specifically designed to elicit original and relevant ideas may have a stronger impact on fostering creativity than their non-creative counterparts or not playing games at all.

Kuo et al. (2023) investigated the impact of having high school students create board games based on their interests and the content of an 18-week civic education course. They found that the process of creating board games led to increased verbal and figural creativity, as well as enhanced civic competencies. This finding suggests that incorporating board game design into educational curricula can promote both creativity and domain-specific learning.

### 5.2. Video Games

Though research on the connection between video games and creativity is relatively limited, initial findings have been promising (Rahimi & Shute, 2021). Blanco-Herrera et al. (2019) conducted an experimental study comparing the effects of playing Minecraft (with or without instructions) to two control conditions: playing a non-creative video game (NASCAR) and watching a TV show (Crocodile Hunter). Results indicated that playing Minecraft without instructions improved graphic creativity compared to both control conditions. However, no effect was found for either verbal divergent or convergent thinking. This finding suggests that certain types of video games, particularly those that promote exploration and unstructured play, may facilitate the development of specific dimensions of creativity, such as graphic creativity.

Experimental studies have explored the impact of various types of video games on divergent thinking. Yeh (2015) found that playing an action video game resulted in enhanced divergent thinking compared to playing a non-action video game. Similarly, Moffat et al. (2017) observed that playing a first-person shooter or a puzzle game led to increased divergent thinking, in terms of flexibility specifically. Though these studies had small sample sizes, they provide preliminary evidence that certain genres of video games may foster creativity.

Other studies have focused on video games explicitly designed to foster creativity. Hsiao et al. (2014) developed a digital game-based learning (DGBL) system, ToES, to enhance both creativity and manual skills in fifth-grade students. In a pre-post experimental study, they compared an experimental group playing ToES to a control group receiving traditional classroom instruction, across the span of a nine-week electrical science class. Results demonstrated notable improvement of divergent thinking in the post-test compared to the pre-test in the experimental condition, whereas the control group did not show differences on divergent thinking performance.

### 5.3. Tabletop Role-Playing Games

In the realm of tabletop role-playing games, Karwowski and Soszynski (2008) developed the Role Play Training in Creativity (RPTC), a creativity training program based on the mechanics of role-playing games. The program was integrated into workshops and resulted in a clear improvement in divergent thinking, particularly in terms of fluency. A replication of the RPTC program (Dyson et al., 2016) yielded similar results on overall divergent thinking. These findings indicate that incorporating role-playing game mechanics into creativity training programs can effectively enhance divergent thinking.

## 6. Discussion

### 6.1. The Framework

This paper proposes a comprehensive framework, based on experiential learning theory and game-based learning, which aims to explain how different forms of games can help promote and improve creativity. The framework is structured using the input-process-output approach. To grasp the whole picture, it is essential to perceive the dynamics between personal attributes, game attributes, and the learning process. The well-known sandbox video game Minecraft, having been studied extensively since its release (García-Álvarez & Acevedo-Borrega, 2025), can provide a clear illustration of such an articulation.

A player’s engagement in Minecraft is first shaped by their personal attributes. For instance, a player high in imagination might be able to envision a complex castle before placing a single block, whereas another player high in playfulness might simply enjoy experimenting with the game’s physics and mechanics, for their own sake. These personal attributes influence how the players perceive and utilize the game’s attributes. Minecraft itself offers numerous attributes that can promote and improve creativity. Its primary affordance is content creation: the game’s world is a procedurally generated canvas where players can build virtually anything that they conceive (Stapleton & Goldstein, 2025). This is further supported by affordances like a high degree of flexibility and a vast explorable and interactive environment, imposing very few restrictions on the player. Minecraft mechanics also inherently demand creativity from neophytes to experienced players (Cipollone et al., 2014). The absence of instructions is key to Minecraft’s gameplay, and as such the game experience is mostly player-directed (Blanco-Herrera et al., 2019; Nebel et al., 2016). This absence of well-defined problems in turn heavily solicits openness to experience and tolerance of ambiguity from the player (creative dispositions) to understand and set goals, as well as requires both divergent thinking and convergent thinking to navigate through the game experience (Thornhill-Miller et al., 2023). The experiential learning process then facilitates the linking of these attributes into a cohesive learning experience (Kolb, 1984). A player might start by exploring the environment (concrete experience), then reflect upon the materials collected (reflective observation). With this information, the player can then formulate a plan that is coherent with their personal attributes, like building a small shelter or even a castle base (abstract conceptualization), and finally, the player can actively try to build it (active experimentation). Through this cycle, the player is not only using the game attributes provided by Minecraft but also involving personal attributes to iteratively and creatively achieve goals and solve problems. This cycle repeats continuously, from building simple shelters to designing complex automated farms or mega-bases. The repeated engagement in this learning cycle leads to creativity benefits and learning outcomes (Kolb, 1984). In the short term, the player may learn specific creative problem-solving skills within the game. In the long term, this sustained practice may lead to a more general enhancement of creative potential. The player may improve their ability to think flexibly, to generate novel ideas and to persist through trial-and-error. This success, in turn, can bolster their creative self-efficacy (i.e., their trust in their ability to be creative in a given situation), by providing mastery experiences and thus revealing that they too can be creative within a particular setting (Bandura, 1997), potentially transferring to challenges and activities outside the game.

### 6.2. Practical Implications

Beyond its theoretical articulation, the G-CEF framework holds value for practice in educational, professional, and design contexts. Educators can employ the G-CEF as an evaluative rubric to select commercial games that will align with pedagogical objectives, allowing for a selection process based on specific game attributes conducive to creativity, rather than subject matter alone. For instance, if a high school teacher wants to improve their students’ creative problem-solving skills, the teacher could select a game that “scores” high on affordances like content creation and tools, and on game mechanics like divergent and convergent thinking. The teacher’s role would then be to facilitate the learning process and guide the scaffolding therein (Molin, 2017; Vygotsky, 1978), aided by the knowledge of the experiential learning stages. Besides selection, the framework can provide guidelines towards the making of tailored game activities based on the means and objectives of the teacher, so that such games empower the role of the teacher in the classroom (Hanghøj & Brund, 2010).

In professional contexts, instructional designers can also utilize the G-CEF as a blueprint to identify or develop serious games or gamified training modules, by mapping learning objectives onto specific game attributes (Arnab et al., 2015): choosing affordances such as open-ended problem spaces or an engaging narrative, or mechanics that encourage originality and mental flexibility, depending on desired creativity outcomes.

For game developers, the G-CEF can serve as a design framework for creating games aimed at promoting or even improving creativity. The list of game attributes provides a concrete set of features for implementations. A developer could design a game with strong affordances for creativity such as a high degree of flexibility, robust content creation systems and a rewarding progression loop. Game challenges could be structured as open-ended problems to encourage novel solutions and foster tolerance of ambiguity (Ulger, 2018). Being cognizant of these attributes can also be used to reverse-engineer effective and popular games, to better understand what makes them potent for creativity.

### 6.3. Limitations

As with all frameworks, the theoretical nature of the G-CEF necessitates acknowledging several limitations that warrant precautions when using it. These limitations notably concern the framework’s generalizability, the potential risks of instrumentalizing play, and the ongoing debate surrounding the efficacy of serious games. 

One limitation of the G-CEF is its potential lack of applicability across different demographic and cultural contexts. Creativity is not a culturally universal concept: what is considered novel or useful can vary significantly between cultures and societies (Niu & Sternberg, 2006). As such, game attributes that foster creativity in one culture may not be as effective in another. For instance, games emphasizing individual achievement and competition might resonate differently in collectivist cultures that prioritize collaboration and group harmony (Hofstede, 2011). Furthermore, the framework’s relevance may differ across age groups. The cognitive and conative factors linking gaming to creativity likely operate differently in children, adolescents, and adults. While pretend play is a known predictor of creativity in children (Russ & Doernberg, 2019), the mechanics that will engage adults may be substantially different, as different generations present distinct preferences and motivations to play games (Bunz et al., 2020; Osmanovic & Pecchioni, 2016; Seçer & Us, 2023).

A second consideration is the inherent tenson between structured, goal-oriented learning and the spontaneous nature of play. The framework proposes using games as a vehicle for creativity enhancement, which risks turning play into a form of work. This instrumentalization of play can lead to over-structuring, which can be seen as detrimental to creative expression. Caillois (2001) distinguished between *paidia*, unstructured and spontaneous play, and *ludus*, rule-based and goal-oriented games. An excessive focus on *ludus*, by emphasizing specific mechanics and learning outcomes, may diminish the freedom and exploration inherent to *paidia*, that are arguably necessary for creative expression and promotion. When applying the G-CEF, for instance in the classroom, a delicate balance is thus required to ensure the learning environment enriches rather than constrains the beneficial play experience.

Finally, the G-CEF operates in the context of ongoing debates regarding the effectiveness of serious games, designed for educational or training purposes (Backlund & Hendrix, 2013; Serrano-Laguna et al., 2018). A recurring critique is that many such “games” are less engaging than their commercial off-the-shelf counterparts, because educational content is infused with gamified elements but ultimately fails to achieve a proper and engaging gaming experience (Silveira & Villalba-Condori, 2022). The experiential learning cycle depends on sustained engagement from the player. If a game designed for creativity training feels forced or uninspired, players are unlikely to achieve the immersion necessary for optimal learning (Hsu & Cheng, 2021). This could mean that commercially successful games, which are designed to maximize player engagement, may be more effective vehicles for creativity enhancement than purpose-built educational games, even if their learning objectives are implicit rather than explicit. Investigating the learning outcomes of engaging with commercial games compared to serious games could help determine if one approach is more effective in the case of creativity.

### 6.4. Future Directions

As with all frameworks, several facets require deeper exploration and refinement to capture the various interactions at play. One avenue for future research is to expand upon and delineate the personal attributes involved in the G-CEF. Initial studies have investigated and confirmed the mediating roles of PsyCap and playfulness (Mercier et al., 2025; Mercier & Lubart, 2023b), but the proposed pathways for other attributes remain to be empirically tested, like imagination, mind-wandering and mindfulness. Furthermore, research is needed to delineate the unique contributions of some conceptually overlapping attributes, like the constructs of mind-wandering and mindfulness, in order to clarify their specific roles in the framework.

A deeper dive into game attributes is also warranted. Different game genres, given their unique design affordances and mechanics, might influence creativity in distinct ways (Stapleton & Goldstein, 2025). Role-playing games, emphasizing narrative and character development, might yield outcomes that contrast with strategy games. Moreover, the alignment with gaming preferences may amplify the creative benefits accrued, emphasizing the importance of player motivation in this equation. The specific contributions of different game attributes require systematic investigation. Experimental studies could help isolate the impact of specific affordances or game mechanics. For instance, a game focused on divergent thinking and originality should induce different learning outcomes compared to a game with mechanics centered around flexibility and convergent thinking. This also denotes the importance of extending and diversifying creativity measurement in experimental studies, beyond traditional divergent thinking tasks (Zhang et al., 2025). It is essential to incorporate measures of more aspects of creativity, such as convergent thinking, creative self-efficacy, or creative mindset. Additionally, comparing digital games with their non-digital counterparts could also show unique avenues of creative enhancement, as the tactile engagement in analog games might influence the learning process differently than the immersive environment of video games.

Finally, one notable and under-investigated avenue concerns the enhancement of collective creativity. Currently, most results published at this time pertain to individual creativity (Zhang et al., 2025). The place of group and team creativity needs to be considered as well. Notably, board games and role-playing games are inherently social activities, as is the case for multiplayer video games (Stapleton & Goldstein, 2025), and it might be the case their effectiveness is even more pronounced when assessing the group’s creative performance, for instance using brainstorming tasks (Bechtoldt et al., 2012).

## Data Availability

No new data were created or analyzed in this study. Data sharing is not applicable to this article.
